# Daridorexant and Insomnia in Clinical Practice: A Nominal Group Technique Consensus Study among Italian Sleep and Insomnia Experts

**DOI:** 10.2174/011570159X367329250717020016

**Published:** 2025-08-06

**Authors:** Luigi Ferini-Strambi, Dario Arnaldi, Enrica Bonanni, Alessandro Cicolin, Gian Luigi Gigli, Claudio Liguori, Carolina Lombardi, Liborio Parrino, Federica Provini, Monica Puligheddu, Andrea Romigi, Rosalia Silvestri, Laura Palagini

**Affiliations:** 1 Sleep Disorders Center, Università Vita-Salute San Raffaele, Milan, Italy;; 2 Sleep Lab, Clinical Neurophysiology, Genova, Italy;; 3 DINOGMI, University of Genoa, Genova, Italy;; 4 Regional Sleep Medicine Centre, Clinical Neurology, AOUP, University of Pisa, Pisa, Italy;; 5 Regional Sleep Medicine Centre, Department of Neuroscience Rita Levi Montalcini, Turin, Italy;; 6 Clinical Neurology, Department of Medicine (DMED), University of Udine, Udine, Italy;; 7 Neurology Unit, University Hospital of Tor Vergata, Rome, Italy;; 8 Department of Systems Medicine, University of Rome Tor Vergata, Rome, Italy;; 9 Sleep Disorders Center Unit, Istituto Auxologico IRCCS, Milan, Italy;; 10 Department of Medicine and Surgery, Milano Bicocca University, Milan, Italy;; 11 Department of Neuroscience, Psychiatric Section, Azienda Ospedaliera Universitaria Pisana (AUOP), Pisa, Italy;; 12 Sleep Disorders Center, University of Parma, Parma, Italy;; 13 Department of Biomedical and Neuromotor Sciences, University of Bologna, Bologna, Italy;; 14 IRCCS, Istituto delle Scienze Neurologiche di Bologna, Bologna, Italy;; 15 Department of Medical Sciences and Public Health, University of Cagliari, Monserrato, Cagliari, Italy;; 16 IRCCS Neuromed Istituto Neurologico Mediterraneo, Pozzilli (IS), Italy;; 17 Departmental Faculty of Medicine, Saint Camillus International University of Health and Medical Sciences UniCamillus, Rome, 00131, Italy;; 18 Sleep Medicine Center, Neurophysiopathology and Movement Disorders Unit, Department of Clinical and Experimental Medicine, University of Messina, Messina, Italy

**Keywords:** Insomnia, daridorexant, orexin, DORA, chronic insomnia disorder, sleep health, insomnia treatment

## Abstract

**Introduction:**

Chronic insomnia disorder significantly affects cognitive, emotional, and physical health. Recently, the dual orexin receptor antagonist (DORA) daridorexant was approved for treating chronic insomnia in several countries. Given the limited evidence available, expert consensus was sought to clarify key clinical issues, inform practice, and guide future research.

**Methods:**

Thirteen Italian sleep experts employed the Nominal Group Technique (NGT) to identify and rank important clinical questions. The process involved independent thought generation, group discussion, and online voting using a 5-point Likert scale.

**Results:**

The NGT process resulted in 55 statements across five key clinical questions, with relevance scores guiding their categorization into three tiers. Key findings highlight daridorexant’s mechanism of action, safety profile, efficacy on night and day parameters, and suitability for long-term use. The experts emphasized cross-tapering strategies for switching from other hypnotics, the importance of sleep psychoeducation, and using the Insomnia Severity Index and sleep diaries for treatment evaluation.

**Discussion:**

Daridorexant may address insomnia without increasing sedation *via* its dual orexin receptor antagonism. Daridorexant seems to be effective and safe even in special patient populations, such as the elderly and those with comorbid conditions (neurodegenerative disorders and cognitive impairment, comorbid insomnia and sleep apnea, psychiatric conditions and mood disorders, epilepsy, and restless leg syndrome), thus representing a new, promising option for insomnia treatment.

**Conclusion:**

The expert consensus provides a comprehensive framework for daridorexant clinical application, advocating for further research to expand the evidence base and refine best practices, as well as underscoring the importance of a multidisciplinary approach that combines both pharmacological and psychosocial interventions to optimize outcomes.

## 1 INTRODUCTION

Insomnia disorder is a prevalent sleep disorder affecting approximately 10% of the adult population worldwide, with another 20% experiencing occasional symptoms. Insomnia is often chronic, with a ∼60% persistence rate over 5 years and frequent relapses in subsyndromal patients [1-3].

Chronic insomnia disorder (as defined by the latest International Classification of Sleep Disorders, ICSD-3-TR), or Insomnia Disorder (as defined by the latest Diagnostic and Statistical Manual of Mental Disorders, DSM-5-TR), is characterized by both nighttime and daytime symptoms occurring ≥3 nights per week for ≥3 months [4-6]. Thus, chronic insomnia disorder is a 24-hour condition, with significant detrimental impact on cognitive abilities, memory, perception, alertness, reaction time, problem solving, fatigue, and daytime sleepiness [2, 7]. Insomnia and sleep disturbances are associated with an increased risk of developing dementia, Alzheimer’s disease [8], cardiovascular conditions [9], mood/emotional state instability, psychiatric conditions such as depression, anxiety, bipolar disorders, and suicide risk and ideation [2, 10, 11]. Such widespread impairments and comorbidities result in high costs for society and healthcare services as well [12, 13]. Effective treatment strategies for insomnia are therefore pivotal in maximizing clinical outcomes, elevating patients’ quality of life (QoL), improving daytime and nighttime symptoms, and reducing the health and economic burden.

So far, the neurobiological mechanisms underlying insomnia involve the role of hyperarousal, the hyperactivation of the stress system at a central and peripheral level, and a deficit in nighttime arousal deactivation [2]. Guidelines for chronic insomnia disorder treatments indicate cognitive behavioral therapy for insomnia (CBT-I) as the first-line approach [14-18]. However, pharmacological options remain the most common and easy-to-access treatment in clinical practice; these include gamma-aminobutyric acid (GABA)-A receptor positive allosteric modulators, melatoninergic receptor agonists, and dual orexin receptor antagonists (DORAs), the latter selectively acting on one of the neurobiological mechanisms of the sleep-wake cycle [14, 17, 19]. Among DORAs, daridorexant has been recently approved in Europe, Switzerland, the UK, the USA, Canada, Japan, and China [20-25]. Daridorexant acts as a DORA (OXR1 and OXR2); several randomized and observational studies showed its efficacy and effectiveness in significantly improving sleep measures and daytime functioning, in both the short- and long-term [26-33], also improving health-related QoL [34]. Guidelines suggest daridorexant as one of the first pharmacological approaches in case CBT-I fails or is not available, and open up the possibility of longer-term treatment due to its safer profile and absence of misuse risk compared to hypnotics [14, 17, 18, 20, 21].

As daridorexant was introduced in Italian clinical practice in 2022, real-life data are still scarce and several questions on its use in day-to-day clinical practice remain open; these comprise which patients’ and insomnia features result in better clinical outcomes, what are the best approaches to optimize treatment, management, and introduction of daridorexant in patients naïve to hypnotics or already undergoing hypnotic treatment, how comorbidities affect treatment, among others consensus methods can be of use in providing an evidence base in situations where evidence is insufficient or lacking; for this reason, we carried out a consensus exercise through the means of the Nominal Group Technique (NGT) [35-37] between Italian physicians who are experts in sleep medicine and have been prescribing daridorexant in their clinical practice. Here, we report our findings in accordance with the ACCORD guidelines on consensus studies (Supplementary Material) [38].

## 2 MATERIALS AND METHODS

### 2.1 Expert Panel

The selection of the expert panel was based on predefined criteria to ensure high-level expertise and experience in the treatment of insomnia and the use of daridorexant in clinical practice. The selection was conducted by invitation, targeting professionals with recognized expertise in sleep-wake disorders, significant clinical experience in managing insomnia and other sleep disturbances, and active prescribing of hypnotic drugs. The panel was formed in March 2024 and included 13 Italian sleep medicine experts, namely 12 neurologists and one psychiatrist, all of whom are directors or senior clinicians at major accredited Italian sleep medicine centers. While not intended to be a comprehensive representation of all Italian experts in insomnia treatment, the panel reflects a broad range of clinical perspectives from multiple regions and leading institutions across Italy. This selection process aimed at providing a well-rounded consensus on the clinical application of daridorexant. Considering all Panelists, we estimated that the number of patients undergoing daridorexant treatment was 350-400 at the time of expert recruitment. LFS (neurologist) and LP (psychiatrist) served as chairpersons and project coordinators. The expert panel sought the help of a methodologist from an independent scientific consultancy agency (Augusto Martellini, Polistudium srl, Milan, Italy) to provide meeting and methodological facilitation, material preparation, and scientific accuracy.

The panel sought to identify some major clinical questions of interest for the clinical practice through internal discussions, clinical experience, and a non-systematic review of the scientific literature on sleep, insomnia, and daridorexant. These questions were used as inputs to generate several statements through the means of the NGT.

### 2.2 Nominal Group Technique, Statement Drafting, and Statement Ranking

The NGT is an expert-based, direct, and structured technique used to manage meetings aimed at making decisions on a specific topic on which there is no strong evidence, and is commonly used in consensus studies [35-37]. The NGT comprises four phases, namely (1) silent generation, (2) thought sharing, (3) statement definition, and (4) statement ranking. During the silent generation process, the experts were asked to independently develop their own thoughts and opinions (*i.e*., “items”) according to the key questions; no discussions or feedback were allowed in this step. All the items (opinions) were collected by the methodologists and shared with the participants during an in-person meeting. During these second and third phases (thought sharing and statement definition), the experts were encouraged to discuss and give feedback on the different opinions.

The facilitator collected all items and experts’ opinions, re-elaborated the items to merge similar ones and give consistency, and finally drafted the preliminary statements. The preliminary statements were then shared with the experts, who had the opportunity to review and/or comment on all items to ensure appropriate wording and content. The final statements were eventually drafted, taking into account all experts’ revisions and opinions, and were then ranked through an online survey.

During the ranking phase, the experts were asked to vote on the statements according to their relevance in clinical practice through the means of a 5-point Likert scale (1 = irrelevant, 2 = somewhat irrelevant, 3 = somewhat relevant, 4 = relevant, 5 = extremely relevant). The voting was carried out online through the use of the online survey platform SurveyMonkey between March and April 2024. For each statement, the relevance scores attributed by each participant were added in order to obtain a cumulative score. The scores thus obtained were subjected to a k-means clustering process using statistical software, with the statements grouped according to each key question. K-means clustering is an unsupervised learning method that partitions *n* observations (in this case, the NGT-derived statements) into *k* clusters, assigning each observation to the cluster with the nearest mean (centroid). In our study, the number of clusters (*k* = 3) was defined *a priori* (*i.e*., subjectively) based on the authors’ judgment, aiming to distinguish between statements of high, moderate, and lower perceived relevance. While the clustering algorithm was applied objectively, the interpretation of clusters as tiers of importance (tier-1, tier-2, and tier-3) was informed by the distribution of cumulative scores and subjectively labeled to reflect their relative clinical significance.

The NGT structured, multi-step, and transparent process allowed us to minimize bias and ensure balanced representation of opinions, overcoming disagreement among experts. Indeed, during the NGT sessions, all participants had an equal opportunity to express their perspectives without external influence, and all the proposed statements were kept and openly discussed in a moderated setting, allowing for clarification and refinement. The statements were then independently rated by every member of the panel according to their own perceived level of relevance/importance; eventually, k-means clustering according to the statements’ cumulative scores ensured that the most widely agreed-upon ones were prioritized. This approach ensured that differing viewpoints were systematically considered and that the conclusions reflected a collective expert consensus rather than individual opinions only.

The final results were shared and discussed among the expert panel in an online meeting held in April 2024. The NGT results and the discussions were used to draft the present paper.

All meetings, discussions, surveys, and other activities were carried out in Italian. The study was not prospectively registered.

## 3 RESULTS

All 13 experts took part in the NGT, voted in the survey, and participated in the subsequent discussion meeting. The experts identified five major clinical questions, which are reported in Table **1**.

The NGT resulted in the drafting of 55 statements: 11 related to the first key question, 15 to the second key question, 12 to the third key question, 7 to the fourth key question, and 10 to the fifth key question. Clustering analysis resulted in three importance clusters (tier 1, tier 2, and tier 3 statements) for every key question, except for key question 4, which stratified into only two clusters (tier 1 and tier 2). Overall, 17 (30.9%) statements were considered extremely relevant (tier 1), 21 (30.9%) relevant (tier 2), and 17 (38.2%) somewhat relevant (tier 3), as all proposed statements were considered at least somewhat relevant; all were kept for discussion. The statements, ordered by key questions and by relevance scores, are reported in Tables **2**-**6** and briefly presented in the following paragraphs. Fig. (**1**) presents a flowchart detailing the management of patients undergoing treatment with daridorexant.

### 3.1 Introduction of Daridorexant into Therapy

The main reason behind the introduction of daridorexant into therapy for chronic insomnia disorder lies in its innovative mechanism of action, which reduces hyperactive wakefulness without increasing sedation. Daridorexant demon strated an optimal safety profile, with a low incidence of adverse events and minimal interactions with other medications, making it suitable for complex patients, such as the elderly or those on polypharmacy. While it avoids common GABAergic-related side effects like tolerance and dependence, daridorexant was recognized as particularly effective in long-term use, showing benefits for sleep and daytime functioning. However, its efficacy in treating various types of insomnia and its role in improving patient beliefs about sleep have been considered of lower relevance (Table **2**).

### 3.2 Introduction of Daridorexant in Non-naïve Patients

The evaluation of current insomnia treatments and the tailoring of the introduction method based on patient type, insomnia severity, and previous treatments were reported as relevant points. Patient awareness on combining psychoeducation with pharmacological therapy was also emphasized. Cross-tapering was reported as the preferred method for transitioning, also for long half-life benzodiazepines (BZs), as it helps avoid withdrawal symptoms. Active consultation between psychiatrists and neurologists for treating patients was deemed interesting but a less relevant strategy. Direct switching was not suggested due to the risk of treatment failure associated with the abrupt discontinuation of the previous therapy, rather than concerns about the efficacy of daridorexant. (Table **3**).

### 3.3 Patient Profiles with Greater Benefits from Daridorexant Therapy in Terms of Clinical Outcomes

Daridorexant was considered particularly beneficial for patients with chronic insomnia disorder comorbid with dementia, epilepsy, obstructive sleep apnea (OSA), and psychiatric disorders, as these conditions do not negatively impact treatment response. It was also considered especially effective in managing nighttime awakenings and insomnia in neurodegenerative syndromes. There was no perceived difference in efficacy between treatment-naïve and non-naïve patients if therapy transition is properly managed, although reasons for non-responsiveness in some patients remain unclear (Table **4**).

### 3.4 Best Practices for Daridorexant Treatment Optimization

Clarifying to patients that sleep regulation is a gradual process through sleep psychoeducation intervention was reported as a highly relevant strategy to optimize daridorexant treatment in clinical practice. Advising patients that the benefits of treatment, such as improved daytime restfulness and better sleep scale scores, occur gradually and not immediately, was deemed crucial. Dedicating time to address patient concerns and using a 50 mg/day dose were also reported as useful methods to optimize the treatment. Other interesting but less relevant interventions, according to the expert’s opinion, were informing patients about the daridorexant mechanism of action or the absence of rebound insomnia or craving in case of discontinuation, as well as the indication to maintain treatment for at least 3 months (Table **5**).

### 3.5 Evaluation of Daridorexant Response

High-relevance methods to evaluate the therapeutic response to daridorexant included sleep diaries and the Insomnia Severity Index (ISI), which was suggested for both clinical and outpatient settings. Objective assessments, such as polysomnography or actigraphy, are generally reserved for cases where there is no response to treatment, and have been reported as less relevant tools in clinical practice. Relying solely on patient-reported outcomes without scales or using tools like the pre-sleep arousal scale has been reported as less effective in routine clinical practice (Table **6**).

## 4 DISCUSSION

The introduction of DORAs to treat insomnia has been a breakthrough within sleep medicine [26-33]. The availability of a new class of drug opens up new scenarios and challenges for insomnia treatment paradigms, especially considering that GABAergic treatments, the most used insomnia drugs in Italy, are not indicated for long-term use [39]. However, empirical data to optimize clinical practice and maximize outcomes with daridorexant - the only DORA approved in Italy - are still few. We carried out a consensus study with NGT to understand the most relevant matters guiding Italian sleep experts’ choices, which are discussed below.

### 4.1 Reasons to use Daridorexant

Daridorexant's mechanism of action was reported as the most relevant reason for introducing the drug in clinical practice. The function of the orexin system is to adapt arousal levels to behavioral needs through the increase of vigilance in response to stress and emotions [40, 41]. An overactive orexin system might act on wake-promoting areas that are not sufficiently inactivated during sleep in people with chronic insomnia disorder [2]. To date, insomnia is primarily associated with hyperactivation of the arousal and stress systems [2, 40]. Although GABAergic modulation is essential for sleep-wake regulation, daridorexant provides a more targeted approach by selectively antagonizing the orexin system, reducing hyperarousal while preserving sleep architecture [20, 27, 42-45].

#### 4.1.1 Safety and Metabolism

BZs and Z-drugs may suffer from safety shortcomings and side effects such as tolerance, dependence, addiction, misuse, rebound insomnia, and withdrawal symptoms [3, 20, 46]. A randomized clinical trial (RCT) demonstrated the efficacy and safety of 12-month daridorexant treatment in over 800 patients; the study showed sustained improvements in nighttime symptoms and daytime functioning, with adverse events comparable to placebo. Additionally, daridorexant did not cause dependence, tolerance, abuse, or rebound effects [31]. This safety and efficacy profile allows for longer-term use in contrast to the conventional four-week insomnia treatment typical of BZs.

Chronic insomnia disorder is not an isolated psychobiological disorder; its chronic nature and its tendency to relapse are probably due to brain network topology alterations such as abnormal connectivity patterns in salience and emotional networks [40, 47-50]. A long-term pharmacological treatment, especially if coupled with behavioral interventions, might be of use in rewiring the brain to a more physiological state.

Daridorexant is metabolized by cytochrome P450 3A4 (CYP3A4), and its concomitant usage with strong CYP3A4 inhibitors is contraindicated in Europe and not recommended in the USA [20]. Pharmacokinetic studies suggest that daridorexant is not a perpetrator of CYP3A4-mediated drug-drug interactions with diltiazem and midazolam [51]; co-administration of daridorexant up to 50 mg with citalopram did not result in serious or severe adverse effects, nor treatment-emergent effects on electrocardiogram parameters, clinical laboratory, and vital signs, and resulted only in minor changes in pharmacokinetic parameters [52].

#### 4.1.2 Complex Clinical Settings

According to the expert panel, the daridorexant safety profile, with few drug-drug interactions, minimal adverse effects even in the long term, and no risk of addiction, tolerance, abuse, or rebound, is more ideal than that of other insomnia medications for use in complex clinical settings. This includes patients with comorbidities and/or those undergoing polytherapy not only for neurological or psychiatric disorders but also for systemic diseases. Thus, the use of daridorexant seems of particular interest in older patients, a population often characterized by frailty, comorbidities, and polytherapy, who have a higher risk profile for sedative-hypnotics use in terms of impaired cognitive functioning [53], reduced mobility and driving skills [54-56], and increased risks of falls [57], and in whom insomnia is more prevalent than in other populations [1]. Of note, the aging-related sleep fragmentation may actually be due to hyperactivation of the orexin system [58], which could be controlled with a DORA. RCT post-hoc analysis comparing ≥65-year-old adults with chronic insomnia disorder with <65-year-old ones suggest that improvements in the nighttime and daytime variables were comparable between the two age groups and that the higher 50 mg dose is necessary to improve daytime functioning, optimize the improvements in sleep onset and maintenance, without any increased risk of adverse events, relapse, nor carry-over effects to the next morning [59].

#### 4.1.3 Other Reasons

According to the panel, there are other interesting reasons in favor of daridorexant. First, its efficacy profile, which allows it to treat all types of insomnia disorders (sleep maintenance insomnia, sleep onset insomnia, and mixed) [27]; this is reinforced by the fact that it is easily framed within sleep psychoeducation, patient-physician therapeutic alliance and that it might improve patients’ dysfunctional beliefs about sleep [32]. Additionally, its 8-hour half-life [60] is considered ideal for treating chronic insomnia disorders compared to other DORAs or hypnotics, as it aligns with the recommended sleep duration [61], offering a balance between effective nighttime treatment and minimal next-day residual effects. Moreover, it provides significant benefits for daytime functioning, even over the long term. As mentioned, chronic insomnia disorder is a 24-hour condition characterized by both nighttime and daytime symptoms with a significant detrimental impact on cognitive abilities, alertness, reaction time, and daytime sleepiness. Daridorexant helps resolve not only nighttime symptoms but also daytime ones [2, 7, 31].

### 4.2 Sleep Health, Psychoeducation, and the Patient-physician Relationship

The pharmacological advantages given by daridorexant must be coupled with other interventions and shrewdness. Psychosocial approaches are of utmost importance and should be carried out before and regardless of the type of pharmacological intervention [14-16]. According to the experts, previous and ongoing insomnia treatment must be re-evaluated and rationalized if needed. Overall, experts agree that establishing a strong therapeutic alliance is essential for managing patients with chronic insomnia disorder. When setting patient expectations, it is important to emphasize the need for patience; based on clinical trials and real-life experience, up to 1 month of daridorexant treatment might be needed before achieving a measurable response.

#### 4.2.1 Introduction of Daridorexant in Patients Undergoing Other Insomnia Treatment

To date, there is no clear recommendation for switching insomnia medications. When switching from different classes of drugs, Watson *et al*. suggest slow taper/cross-taper BZs, trazodone, mirtazapine, tricyclic antidepressants, and quetiapine; taper and then wait 1-2 days in case of zolpidem and eszopiclone; and direct switch in case of zaleplon and daridorexant [62]. Cross-taper is defined as a gradual reduction of the first insomnia drug while a new insomnia medication is introduced at a low dose and gradually increased, but no clear consensus as to what the tapering schedule should be is currently available in the literature [62]. Many studies report on reducing BZs and Z-drug dose by ~10-25% decrements at intervals of one to several weeks [62, 63], an approach carried out when cross-tapering with daridorexant in real-world practice as well [32]. According to the panel, several factors must be taken into consideration when introducing daridorexant in patients undergoing other chronic insomnia disorder treatments, such as the patient, the severity and type of insomnia, the duration and type of previous treatment.

##### 4.2.1.1 Cross Tapering and “Delayed” Cross Tapering

**Fig. (2):**
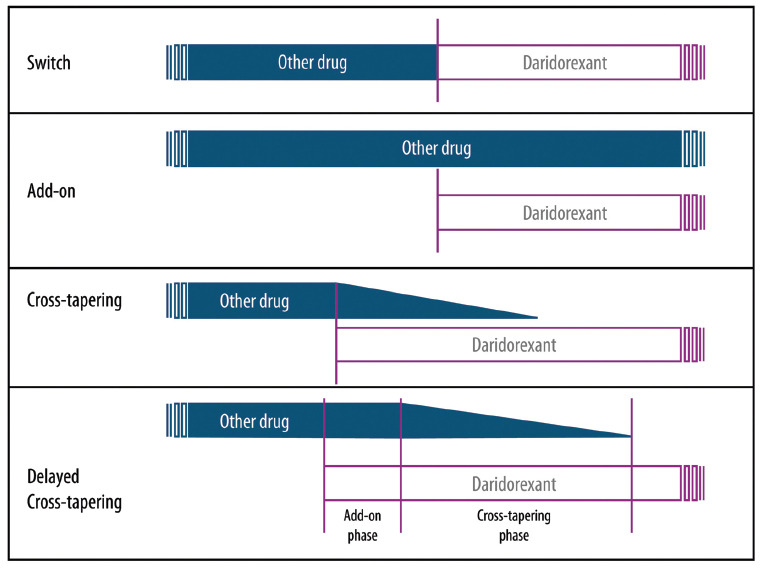
Different ways of introducing daridorexant in patients undergoing other insomnia treatment.

Although cross-tapering regimens usually expect new drugs to be introduced at a low dose and gradually increased, the panel strongly suggests introducing daridorexant already at the full dose of 50 mg; indeed, this dose is the one recommended by the European Medicines Agency as well [21]. One alternative way is to carry out a “delayed” cross-tapering by introducing daridorexant 50 mg as an add-on for a certain timespan so as to allow it to exert its therapeutic effect before starting to taper the concomitant drug(s) (Fig. **2**). Reasons to start daridorexant at 50 mg dose reside in the experts’ personal experiences, confirming the results of clinical studies which suggest that the 50 mg regimen improves sleep and daytime functioning better than the 25 mg [27, 31], especially in older patients [59], with no significant trade-off for safety [20, 26, 59]. The reasons to cross-taper are several and are mainly related to the aim of rationalizing previous treatment, allowing the discontinuation of other insomnia drugs while avoiding adverse effects such as rebound insomnia or other withdrawal symptoms.

##### 4.2.1.2 Switch and add-on

The direct switch can result in a concrete risk of rebound/withdrawal symptoms of the current hypnotics to be tapered off, as well as in treatment discontinuation of daridorexant by the patient [46]. Direct switch may be considered only in rare scenarios, such as extremely motivated patients, but cross-tapering and add-on should be preferred. Using daridorexant as an add-on can, in fact, be sometimes considered, especially during periods of insomnia recrudescence (Fig. **2**).

#### 4.2.2 Duration and Discontinuation of Daridorexant Treatment

Regarding treatment duration and discontinuation, studies and expert experience suggest that treatment, although generally effective after a couple of weeks, should be continued for at least three months to exert long-lasting benefits [27, 32]. Sudden daridorexant discontinuation can be carried out as the drug seems not to be associated with withdrawal and rebound symptoms [27, 31, 62, 64], in contrast to hypnotics [46].

### 4.3 Profiles of Patients with Chronic Insomnia Disorder who Benefit most from Daridorexant

Daridorexant is a relevant therapeutic option for the treatment of chronic insomnia disorder patients with comorbidities and/or under polytherapy. Indeed, the panel underlined the “utility” of daridorexant in terms of (1) ease of management (limited drug-drug interactions and adverse effects, fixed dosages, lack of titration), and (2) robust treatment response, irrespective of comorbidities. Individuals of older age, or those with dementia/cognitive impairment, OSA syndrome, or psychiatric conditions, were reported as interesting candidates for daridorexant treatment. Among these, the highest relevance was given to chronic insomnia in patients suffering from neurodegenerative disorders, followed by “comorbid insomnia” (non better specified), comorbid insomnia and OSA (COMISA; both relevant statements), and then insomnia in patients who have epilepsy, psychiatric syndromes, mood disorders, and restless leg syndrome (all somewhat relevant).

#### 4.3.1 Patients with Other Neurological or Psychiatric Comorbidities

Orexin neurons widely project to several brain areas that can be affected by neurological and psychiatric conditions; preclinical studies seem to suggest that when orexin is antagonized, beneficial effects can arise, possibly due to the direct effect of orexin antagonism and the indirect effect of orexin-mediated modulation of sleep and wake [65]. Clinical data on this matter is scant yet somehow promising. A retrospective study by Fernandes *et al*. [33] enrolled 69 patients starting 50 mg daridorexant, of whom ∼50% presented at least one comorbidity, and >23% presented at least two; the most common ones were depression, anxiety, epilepsy, and mild cognitive impairment (MCI); moreover, most patients were undergoing other treatments. At one month follow-up, there were no differences in subjective and objective outcome measures according to the number and type of comorbidities and previous medications. However, the number of enrolled patients was small [33]. Older patients with MCI often suffer from sleep impairment [66], and these findings substantiate previous data on the possible role of downregulation of orexin neurotransmission to improve sleep in this population [67, 68]. Recently, a rationale for the orexin receptor block in epilepsy has been proposed [69]; in this perspective, the beneficial effects of daridorexant may suggest new therapeutic targets for improving sleep and epilepsy in those with both conditions, even if definite conclusions are yet to be fleshed out.

Palagini *et al*. enrolled 66 patients with chronic insomnia disorder, with most of them suffering from comorbid psychiatric conditions such as depression, anxiety, substance use disorders, and taking concomitant medication [32]. Daridorexant treatment resulted not only in improved sleep outcomes measured with several scales, but also in improvements in depressive, anxiety, and mania symptoms and suicidal ideation [32]. This promising data needs to be confirmed in independent cohorts.

In conclusion, as the orexin system is emerging as interfering with pathophysiological mechanisms at the basis of dementia and Alzheimer’s disease, depression, and lack of emotion modulation, daridorexant might be a valuable means in the treatment of psychiatric and neurological comorbidities [32, 33, 65].

#### 4.3.2 Obstructive Sleep Apnea

Regarding OSA syndrome, it is often associated with insomnia-related symptoms; namely, 30-50% of patients with OSA report clinically significant insomnia symptoms, and 30-40% of patients with insomnia have co-morbid OSA. However, several insomnia medications may impair respiration, and should be used with caution in patients with OSA or respiratory disorders [70, 71]. COMISA is a debilitating disorder that significantly impairs sleep, daytime functioning, and quality of life [72]. One RCT in 28 patients with mild to moderate OSA found that repeated-dose administration of 50 mg daridorexant did not impair nighttime respiratory function based on apnea/hypopnea index and peripheral oxygen saturation, regardless of OSA severity, and was associated with sleep improvement despite the absence of coexisting chronic insomnia disorder symptoms [70, 71]. A subsequent trial with a comparable design in 16 patients with severe OSA corroborated the previous findings. The trial showed improved sleep and no concern for safety, with no differences between daridorexant and placebo for the total number of apneas and hypopneas (suggesting no switch from apneas to hypopneas or *vice versa*), and no effect on the longest and mean duration of respiratory events and the lowest and mean peripheral oxygen saturation [73]. Taken together, these studies suggest that daridorexant could improve COMISA; by targeting insomnia, it may be possible to improve sleep symptoms without exacerbating OSA.

#### 4.3.3 Other Considerations

Finally, the experts also highlighted that they could not identify clear correlations between daridorexant benefits and several other parameters (insomnia duration and type, number of previous treatments, comorbidities, *etc*.); the panel discussions on everyday clinical practice highlighted that in some cases, patients who were anticipated to respond did not, while others with low expectations exhibited satisfactory outcomes.

The Expert Panel's final message is to introduce daridorexant if indicated, as no clear phenotypes are inhibiting the function of the drug.

### 4.4 Evaluating Response to Treatment

The evaluation of an adequate patient’s response to treatments allows physicians to intervene in the treatment schedule promptly. When enquired on the matter, the panel judged the sleep diary and the ISI to be the most relevant instruments. Sleep diaries, especially if standardized, such as the consensus sleep diary [74], are recommended for the diagnosis of insomnia and are used to evaluate treatment response, at least in the literature [2, 15, 19]. Nonetheless, the experts judged as somewhat relevant the statement indicating sleep diary as a “difficult”-to-use instrument, as many patients do not use it consistently. This suggests that, although acknowledging its importance, it might be hard to implement its use in everyday clinical practice. The ISI, on the other hand, might be a viable complement due to its shortness and ease of administration. The ISI was shown to have adequate internal consistency, to be a reliable self-report measure to evaluate perceived sleep difficulties, measures of fatigue, quality of life, anxiety, and depression. Also, it is a valid and sensitive measure to detect changes in perceived sleep difficulties with treatment; changes within ISI scores are significantly correlated with changes in sleep diary measures, polysomnographic parameters, and clinicians’ parallel ISI measures [75-77]. Indeed, the expert panel judged ISI to be more relevant than sleep diaries in the diagnostic phase, and the fact that it should always be performed, even in an ambulatory setting, is extremely relevant, as also suggested by another expert panel [78]. The widespread introduction of the ISI for diagnosis, screening, and evaluating treatment response will allow pooling data of several patients with chronic insomnia disorder undergoing many different interventions and will be needed to compare treatment outcomes in large cohorts, providing important real-world evidence. Sleep diaries should still be used as they are able to provide valuable insights into the patient’s specific condition, duration, distribution of sleep, and sleep-wake cycle across the 24 hours, and response to therapy, helping to guide the best therapeutic approach. The ISI and sleep diary should complement one another.

#### 4.4.1 Polysomnography and Actigraphy

Objective, instrumental studies (namely polysomnography and actigraphy) are currently not recommended to evaluate insomnia response, although literature recently evaluated the use of actigraphy in insomnia diagnosis and treatment [79]. In particular, actigraphy is usually performed when another sleep-wake disorder is suspected, and polysomnography should be performed in case of the suspect of concomitant OSA or periodic limb movement disorder. This is in line with the European Insomnia Guidelines which strongly recommend actigraphy only in case of clinical suspicion of irregular sleep-wake schedules or circadian rhythm disorders, and polysomnography only in case of treatment-resistant insomnia, possibly due to concomitant other sleep-wake disorders (such as periodic limb movement disorder, sleep apnea or narcolepsy), or when large discrepancy between subjectively experienced and polysomnographically measured sleep is suspected [2, 15].

### 4.5 Study Limitations

This study has several limitations that should be acknowledged. First, the statements generated were not based on a systematic review of the literature but rather on a non-systematic review of available studies and experts’ clinical experience, which may have introduced selection bias and missed relevant data; furthermore, there was no grading of the evidence and bias assessment. As such, the statements and their level of importance should not be considered as clinical recommendations or guidelines, but just the shared opinions of the expert panel, which stem from both objective clinical data and personal experience. The study also focused solely on Italian sleep medicine experts, possibly introducing regional bias and limiting the applicability of the findings to international clinical practice. While the study draws on both clinical studies and real-world experience, the variability in real-life clinical practice, regulatory status, and treatment reimbursement could affect the generalizability of the results. Lastly, the focus on daridorexant may limit broader insights into how DORAs in general compare to other available treatments for chronic insomnia.

## CONCLUSION

The consensus among sleep experts indicates that the innovative mechanism of action of daridorexant, which reduces hyperactive wakefulness without increasing sedation, is a critical factor for its use in clinical practice. This mechanism allows for a targeted approach to treating chronic insomnia disorder, addressing the hyperarousal underlying the disorder while relatively preserving sleep architecture.

Clinical trials and real-world evidence reported that daridorexant effectively improves both nighttime and daytime symptoms of insomnia, even in patients with comorbidities such as neurodegenerative disorders, psychiatric syndromes, and OSA. The drug's safety profile, characterized by minimal adverse events and limited drug-drug interactions, makes it a suitable option also for a broad patient profile, including the elderly, those with complex medical histories, and those undergoing polytherapy.

Best practices for the introduction and management of daridorexant therapy were highlighted, emphasizing the importance of sleep psychoeducation and a strong patient-physician therapeutic alliance. These practices include cross-tapering or delayed cross-tapering strategies when switching from other hypnotics to daridorexant and maintaining the treatment for at least three months to achieve sustained benefits. The expert panel also supports the use of the ISI and sleep diaries as primary tools for evaluating treatment response in both clinical and research settings.

In conclusion, daridorexant represents a significant advancement in the therapeutic arsenal for insomnia due to its distinct characteristics compared to sedative-hypnotics, especially in terms of its safety profile and suitability for long-term use. Further research and real-world studies are encouraged to continue refining the optimal use of daridorexant and to expand the evidence base supporting its benefits across different clinical scenarios.

## AUTHORS’ CONTRIBUTIONS

The authors confirm their contribution to the paper as follows: Study conception and design: LFS, LP; collection and interpretation of data: all authors; statistical analysis: LG; manuscript drafting: FP (medical writer); manuscript editing: all authors. All authors reviewed the results and approved the final version of the manuscript.

## Figures and Tables

**Fig. (1):**
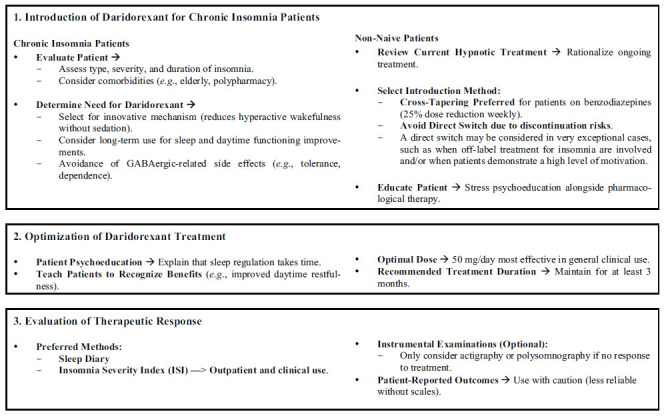
Flowchart outlining the management approach for patients treated with daridorexant, including initiation strategies, optimization of the treatment, and evaluation of the therapeutic response.

**Table 1:** The five key questions used to draft the NGT statements.

1. What were the reasons why you chose to introduce daridorexant for chronic insomnia disorder treatment into clinical practice?
2. Based on your experience, how is daridorexant introduction managed in patients affected by chronic insomnia disorder already being treated with other hypnotic drugs?
3. With respect to the clinical profile of the patient with chronic insomnia disorder (*e.g*., type of insomnia, duration and severity of the disease, comorbidities, previous treatments, and other characteristics are there any profiles which emerged as having benefited more than others from daridorexant therapy in terms of clinical outcomes?
4. What are the best clinical practices to optimize outcomes with daridorexant therapy for the treatment of chronic insomnia disorder?
5. In clinical practice, how have you been evaluating the patient's therapeutic response, and what is the most feasible and effective way to evaluate the chronic insomnia disorder patient's response to daridorexant therapy?

**Table 2:** Statements on reasons to introduce daridorexant.

**1. Why did you choose to introduce daridorexant into therapy?**	
**Statement**	**NGT Score**
**High Relevance**	
Daridorexant has an innovative mechanism of action that does not increase sedation but reduces the hyperactive wakefulness state in patients with chronic insomnia.	62
**Medium Relevance**	
Daridorexant has an optimal safety profile due to the reduced number of adverse events and minimal interaction with other medications.	56
Daridorexant allows long-term therapy even beyond four weeks.	55
Daridorexant can be used in complex patients such as the elderly, those with comorbidities, and those on polypharmacy.	56
Treatment with daridorexant avoids side effects related to GABAergic treatment, such as tolerance, physical dependence, and abuse.	57
Daridorexant can be used in patients with a higher risk profile compared to the general population for GABAergic drugs (*e.g*., elderly patients).	53
**Low Relevance**	
Daridorexant has an efficacy profile that allows for the treatment of all types of chronic insomnia (initial, maintenance, terminal, and mixed).	44
Daridorexant shows significant efficacy on both sleep and daytime activities, even in the long term.	51
The terminal half-life of the drug is 8 hours, ideal for the treatment of chronic insomnia.	48
Early evidence in psychiatric settings suggests daridorexant can improve patients' incorrect beliefs about sleep over time, increasing their ability to manage sleep.	44
Daridorexant fits well within the physician-patient therapeutic alliance, as it pairs well with sleep psychoeducation.	47

**Table 3:** Statements on how to introduce daridorexant in non-naïve patients.

**2. According to your clinical experience, how do you manage the introduction of daridorexant in patients already treated with other hypnotic drugs?**	
**Statement**	**NGT Score**
**High Relevance**	
Before introducing daridorexant, it is important to evaluate and rationalize the current insomnia treatment if necessary.	61
The method of introducing daridorexant in patients already on other hypnotic drugs depends on the patient type, severity and type of insomnia, and duration and type of previous treatment.	57
When introducing daridorexant, it is crucial to explain to the patient the importance of both sleep psychoeducation and pharmacological treatment.	63
If cross-tapering is used to introduce daridorexant, it is important to taper the concomitant drug according to guidelines (25% of the dose each week).	55
**Medium Relevance**	
The introduction of daridorexant is done through cross-tapering, starting from 25 mg of daridorexant and reaching 50 mg or directly introducing 50 mg of daridorexant.	50
Daridorexant is introduced as an add-on.	43
In the case of introducing daridorexant in psychiatric patients, it is advisable for the neurologist to consult with the psychiatrist to evaluate the need to modify ongoing therapies to optimize treatment and manage psychiatric comorbidities.	46
It is advisable to introduce daridorexant through cross-tapering in case of treatment with long half-life benzodiazepines.	51
Introducing daridorexant through cross-tapering helps rationalize treatments, allowing the gradual discontinuation of other sleep medications and avoiding side effects related to abrupt withdrawal (*e.g*., rebound insomnia and withdrawal symptoms).	53
It is advisable to introduce daridorexant through add-on therapy in case of insomnia relapses.	42
It is not advisable to introduce daridorexant through direct switch from another hypnotic drug.	51
Introducing daridorexant through direct switch may pose a real risk of treatment discontinuation by the patient.	50
**Low Relevance**	
The introduction of daridorexant is done through direct switch.	22
In the case of short half-life hypnotics or off-label drugs commonly used for insomnia, it is advisable to introduce daridorexant through direct switch.	29
Direct switch introduction of daridorexant is advisable only for very motivated patients who are aware of the therapeutic path to follow.	36

**Table 4:** Statements on phenotypes responding better to daridorexant.

**3. Considering the clinical condition of the patient with chronic insomnia (*e.g*. type of insomnia, duration and severity of the disease, comorbidities, previous treatments, *etc*.), have specific patient profiles shown greater benefits from daridorexant therapy in terms of clinical outcomes?**	
**Statement**	**NGT Score**
**High Relevance**	
Daridorexant is very useful in patients with comorbidities and difficult to treat (*e.g*., elderly, patients with dementia, epilepsy, sleep apnea, psychiatric patients) as comorbidities do not seem to affect treatment response.	56
There are no differences in efficacy between naïve and non-naïve patients as long as the therapy change is managed correctly.	52
Daridorexant is particularly effective in patients with night-time awakenings who have difficulty falling back asleep.	52
Daridorexant is effective in chronic insomnia comorbid with neurodegenerative syndromes.	54
There are some patients in whom daridorexant is not effective, but the reasons are not yet understood.	54
**Medium Relevance**	
Daridorexant is effective in comorbid insomnias.	50
Daridorexant is effective in chronic insomnia associated with obstructive sleep apnea syndrome.	50
**Low Relevance**	
here do not seem to be clinical phenotypes that respond better/worse to daridorexant.	47
Daridorexant is effective in chronic insomnia associated with epilepsy.	44
Daridorexant is effective in insomnia resulting from psychiatric disorders.	47
Daridorexant is effective in chronic insomnia associated with mood disorders where it seems to improve depressive and mixed symptoms.	46
Daridorexant is effective in chronic insomnia associated with restless leg syndrome.	45

**Table 5:** Statements on best practices for daridorexant treatment optimization.

**4. How to optimize daridorexant treatment in clinical practice? (Best practices for treatment optimization)**	
**Statement**	**NGT Score**
**High Relevance**	
To optimize daridorexant treatment, it is important to carry out sleep psychoeducation, explaining that sleep is a complex biological process that takes time to regulate.	65
To optimize daridorexant treatment, it is important to teach patients to recognize its benefits even if they are not immediately evident (*e.g*., feeling more rested and less irritable during the day, obtaining better scores on sleep scales, *etc*.).	59
To optimize daridorexant treatment, it is important to dedicate time to the patient, listening and answering their questions.	59
Daridorexant can be used at a dose of 50 mg/day, which has been shown to be the most effective in multiple clinical scenarios, unlike 25 mg/day, which is only useful in specific cases.	60
**Medium Relevance**	
To optimize daridorexant treatment, it is important to inform the patient about the innovative mechanism of action and how sleep works.	57
Daridorexant can be discontinued abruptly without problems of rebound insomnia or craving.	54
It is advisable to maintain the treatment for at least three months.	55

**Table 6:** Statements on how to evaluate response to daridorexant.

**5. In your clinical practice, how have you evaluated the therapeutic response of the patient, and what do you consider the most feasible and effective way to evaluate the patient's response to therapy?**	
**Statement**	**NGT Score**
**High Relevance**	
To evaluate the therapeutic response to daridorexant, it is appropriate to use a sleep diary.	50
To evaluate the therapeutic response to daridorexant, it is appropriate to use the Insomnia Severity Index (ISI).	54
The Insomnia Severity Index (ISI) should always be performed, even in an outpatient setting.	54
**Medium Relevance**	
Instrumental examinations have a stronger rationale for research purposes than for clinical practice.	45
Evaluating the response to daridorexant treatment through objective parameters of instrumental exams (polysomnography and actigraphy) is not necessary unless there is a lack of response.	47
It is appropriate to perform actigraphy when there is no expected response to treatment.	45
**Low Relevance**	
The most reliable way to assess therapeutic response is the patient-reported benefit without using scales.	40
To evaluate the therapeutic response in an outpatient setting, it is important to pay attention to what the patient reports, as it is often impossible to use validated scales or instruments.	42
The sleep diary is a difficult tool for evaluating therapeutic response as many patients do not fill it out.	38
To evaluate the therapeutic response to daridorexant, it is appropriate to use the Pre-Sleep Arousal Scale (PSAS).	42

## Data Availability

The data and supportive information are available within the article.

## LIST OF ABBREVIATIONS

BZ: Benzodiazepine
CBT-I: Cognitive Behavioral Therapy for Insomnia
COMISA: Comorbid Insomnia and Sleep Apnea
CYP3A4: Cytochrome P450 3A4
DORA: Dual Orexin Receptor Antagonist
GABA: Gamma-aminobutyric Acid
ISI: Insomnia Severity Index
NGT: Nominal Group Technique
OSA: Obstructive Sleep Apnea
OXR: Orexin Receptor
QoL: Quality of Life
RCT: Randomized Clinical Trial

## STANDARDS OF REPORTING

ACCORD (Accurate Consensus Reporting Document) guidelines were followed.
